# Rapidly fatal Askin's tumor: a case report and literature review

**DOI:** 10.11604/pamj.2014.18.104.4549

**Published:** 2014-05-30

**Authors:** Mustapha Laine, Ismail Abderrahmani Ghorfi, Dalal Lambatten, Fouad Kettani, Ahmed Abid

**Affiliations:** 1 Department of Pneumology, Mohamed V Military Teaching Hospital, Faculty of Medicine and Pharmacy, Mohamed V University, Rabat, Morocco; 2 Institute of Pathology Nations Unies, Rabat, Morocco

**Keywords:** Askin tumor, chest wall, primitive neuroectodermal tumor, Ewing sarcoma, malignancy

## Abstract

An 18-year-old male presented with a mass in the right anterior chest wall. Chest Computed tomography revealed a heterogenous mass of 19X13 cm in the right hemithorax with areas of necrosis. There was associated pleural effusion and infiltration of the soft parts of the chest wall. Bronchoscopy showed a tumor in middle lobe bronchus. CT guided biopsy of the mass was performed. Histological examination showed small round tumor cells with scanty cytoplasm, the nuclei are large and hyperchromatic. The tumor cells were positive for CD99 and neuron specific enolase, negative for cytokeratin, leukocyte common antigen and myogenin. Based on these histologic and immunohistochemical findings, the diagnosis of askin's tumor was made. The extension assessment was negative and the patient was given chemotherapy. Two months later, our patient died. Askin's tumor is a rare, highly malignant tumor affecting children and young adults. It is classified as primitive neuroectodermal tumor of the thoracopulmonary region. Prognosis remains poor. In our case, several prognostic factors may explain the shirt ‘term survival, despite no distant metastasis were found: important tumor size, impossibility of surgical treatment and pleural effusion.

## Introduction

Askin in 1979 [1], described for the first time a malignant and aggressive tumor of the chest wall, affecting young patients. Since, this tumor is known as Askin tumor, and very rare cases are reported worldwide. This tumor is at present classified as primitive neuroectodermal tumor (PNET) of the thoracopulmonary region and strikes by its histological, immunohistochemical and cytogenetic similarities with Ewing′s sarcoma [2]. To date, no treatment is codified and prognosis remains dark. We report a case of Askin tumor in an 18-year-old male and review the different data from the literature.

## Patient and observation

**Figure 1:**
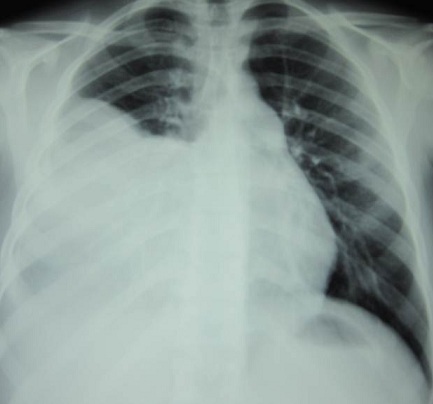
Chest radiograph showing a homogenous opacity in the right hemithorax

**Figure 2:**
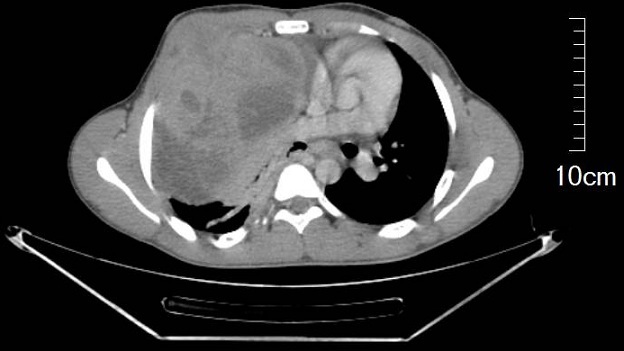
Contrast-enhanced chest CT showing a heterogenous mass in the right lung with infiltration of the soft parts of the chest wall

An 18 years old male presented with a five months history of left chest pain, cough and dyspnea. He had previously been in good health. Physical examination revealed a mass in the right anterior chest wall, dull percussion note and reduced breath sounds over the right lower thorax. There was no lymphadenopathy and patient was in good condition. The chest x-ray revealed homogenous opacity in the right hemithorax (Figure 1). Chest CT imaging (Figure 2) showed a heterogenous mass of 19X13 cm in the right hemithorax with hypodense areas suggestive of necrosis. There was associated pleural effusion and infiltration of the soft parts of the chest wall. The compression of the mediastinum was evident and no rib invasion or mediastinal lymphadenopathy was observed.

Biological tests founds an inflammatory syndrome with elevated C-reactive protein (CRP), plasmatic levels of lactic deshydrogenase (LDH) and neuron specific enolase (NSE) were high. Cytology of the hemorrhagic pleural fluid did not reveal the presence of malignant cells. Bronchoscopy showed a tumor in middle lobe bronchus, histological examination of biopsies was inconclusive.

**Figure 3:**
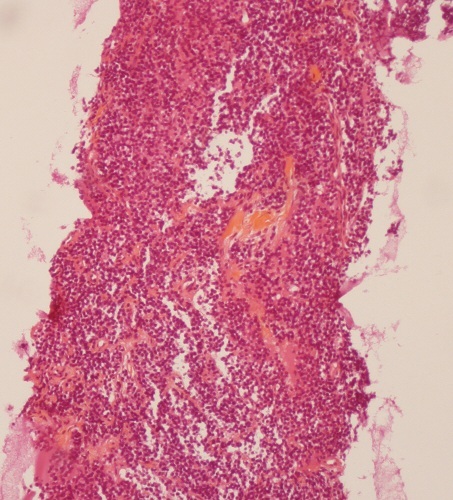
Histological examination (HES, X 100): small round tumor cells with scanty cytoplasm. The nuclei are large and hyperchromatic

**Figure 4:**
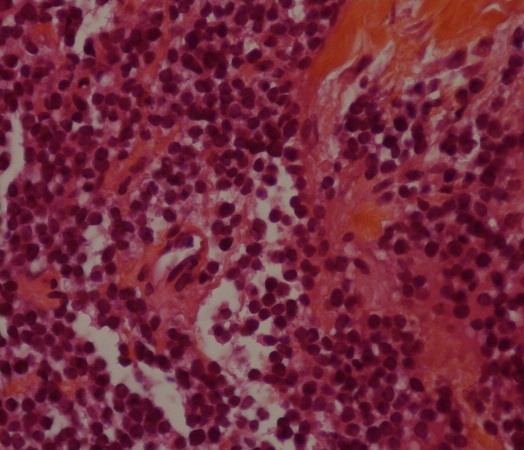
Histological examination (HES, X 400): small round tumor cells with scanty cytoplasm in a rare fibrous stroma. The nuclei are large and hyperchromatic

**Figure 5:**
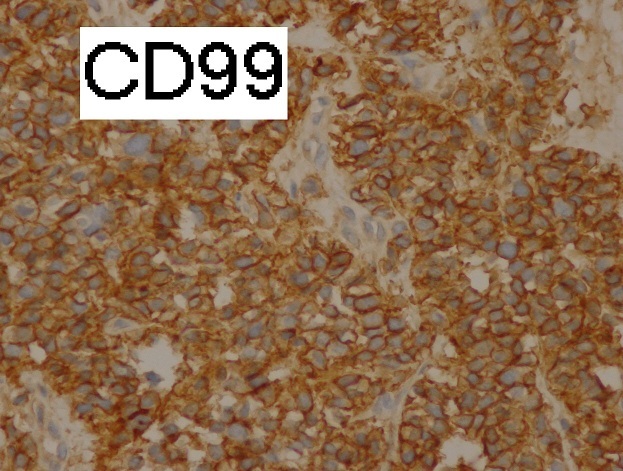
Immunohistochemistrye (immunostaining X 400): diffuse and strong positivity for CD99

**Figure 6:**
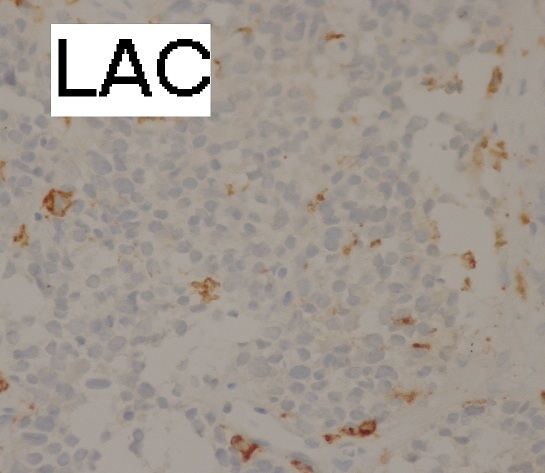
Immunohistochemistry (immunostaining X 400): negativity for LAC

CT guided biopsy of the mass was performed, microscopic examination (Figure 3, Figure 4) showed small round tumor cells with scanty cytoplasm in a rare fibrous stroma. The nuclei are large and hyperchromatic. Mitotic activity is high. The immunohistochemistry study was strongly positive for CD99 (Figure 5) and NSE, but was negative for cytokeratin (CK), leukocyte common antigen (LCA) (Figure 6) and myogenin. Based on immunohistochemistry findings, the diagnosis of Askin tumor was made. Tumor staging procedure, including bone scintigraphy, abdominopelvic and brain CT, did not detect distant metastasis. The patient was given carboplatin-etoposide chemotherapy and has complete 2 cycles of treatment. His physical status worsened. The patient died 2 months after diagnosis, mainly attributed to tumor progression.

## Discussion

Askin′s tumor is a highly malignant tumor of the chest wall from the group of peripheral neuroectodermal tumors. It was first described by Askin in 1979 as a malignant small round cell tumor [3]. Askin′s tumor usually occurs in children, adolescents and young adults, but can develop at any age, with a female predominance in about 75% of cases [4].

The common presentation in patients with askin's tumor is chest pain, respiratory distress or a chest wall mass [3]. Rarely, It may present as pathological fracture or metastasis related symptoms [5]. General symptoms like fever, anorexia and weight loss may occur. Other modes of revelations are even rarer. The biological profile has rarely been studied, in this case the plasma levels of LDH and NSE are high.

The characteristic CT picture in the patient with Askin tumor is that of a heterogenous mass of chest wall origin with areas of necrosis and hemorrhage with or without an intrathoracic component. The MRI shows the same images as CT and help to evaluate the mediastinal, pleural invasion and extrathoracic extension [6]. Tumor extension to pleura, as found in the index case, is associated with effusion. Calcification and lymphadenopathy are rarely seen in these tumors. Distant metastasis spreads to lungs, bones, bone marrow, liver and brain [3].

In our literature review, most cases of Askin's tumor were presented as a mass in the chest wall with or without pulmonary involvement, and the diagnosis was made by biopsy from the mass. Bronchoscopic findings are rarely described. The disease is diagnosed by histologic and immunohistochemical analysis. Cytologic smears of the tumor reveal small round malignant cells that contain little cytoplasm and are arranged in rows. The typical feature is the presence of Homer-Wright rosettes with various layers of cells with fibrillary material [7]. Under immunohistochemical examination, the tumor is positive for several neural markers, such as NSE, CD99, and vimentin [7]. The differential diagnosis includes neuroblastoma, lymphoma, small-cell carcinoma, rhabdomyosarcoma, monophasic synovial sarcoma, and desmoplastic small round cell tumor [8].

Treatment of Askin's tumor should be multimodal, requiring discussion in multidisciplinary tumor working groups. The treatment includes chemotherapy, radiotherapy and surgery. The treatment of an Askin tumor should aim to control local disease and distant metastasis. Thus, the prevailing treatment of an Askin tumor is a combination of neoadju¬vant chemotherapy, radical surgical resection and adjuvant chemotherapy and radiotherapy. Several studies have proved that this aggressive therapy may lead to a longer relapse free survival [3]. Surgical therapy has the most important implication and complete surgical resection is associated with a survival advantage [5]. Neoadjuvant chemotherapy provides better local control of the disease, a less extensive surgery and treats microscopic distant metastasis [9]. Current protocols of chemotherapy are based on a combination of two to six anticancer drugs from doxorubicin, actinomycin D, cyclophosphamide, ifosfamide, vincristine, etoposide, busulfan, melphalan and carboplatin [2]. In our case, Surgery was recused due to the anatomical complexities. The patient was given carboplatin-etoposide chemotherapy. The prognosis of Askin's tumor is very poor. Askin reported that 14 of 18 patients with known diagnosis died 4 to 44 months after diagnosis, and the mean survival period was 8 months [1]. Our patient died 2 months after diagnosis. In a review of 104 patients with askin's tumor, Siddharta et al. concludes that important prognostic factors influencing outcome are: Primary tumor size, pleural effusion, response to chemotherapy, and optimal radiotherapy [10]. Several authors assert that Surgical therapy has the most important implication and complete surgical resection is associated with a survival advantage [5].

In the index case, despite no distant metastasis were found at diagnosis, the patient died 2 months later. The reasons of this shirt-term survival may be important tumor size, impossibility of surgical treatment, and pleural effusion. We believe that this prognosis would have been better if the diagnosis was made earlier, allowing surgical excision. With this case report we hope to highlight the importance of keeping in mind askin tumors as one etiologic possibility of chest wall tumor. Early diagnosis could improve the prognosis.

## Conclusion

The diagnosis of Askin's tumors is difficult because of their rarity and their clinical polymorphism. It requires immunohistochemical workup supported by imaging investigations. Treatment is not yet codified. However, surgery is predominant. The Prognosis is poor. We describe a case of a 18 year old male without metastatic disease at presentation. Despite chemotherapy, the patient died 2 months later. Several prognostic factors may explain this shirt-term survival. We speculate that surgical therapy has the most important implication in the outcome of patients with Askin's tumor.
